# Report on Practical Surgery

**Published:** 1858-05

**Authors:** Edward Hartshorne

**Affiliations:** One of the Surgeons of Wills’ Hospital, Philadelphia


### REPORT ON PRACTICAL SURGERY FOR THE YEAR 1857.

By EDWARD HARTSHORNE, M.D., one of the Surgeons of Wills' Hospital, Philadelphia.

THE unavoidably short time allowed, on this first occasion, for the examination of the journals and society transactions of the past year, required in the preparation of a review of the more important contributions to the literature of the practical surgery of the period, has rendered it impossible to present a satisfactory notice of much that might with advantage be included under such a head. Want of space also, as well as want of time for the necessary condensation, has obliged the reporter to pass over many articles of value, and to confine himself strictly to the analytical exhibition of the most available portions of such papers only as appeared to possess the greatest practical importance, or were essentially novel or rare in the nature of their topics. Many subjects have been omitted altogether, to be taken up separately in a future number, or in other periodical reports; while the consideration of elaborate monographs, such as the well-known papers of Dr. F. Hamilton on Fractures, has to be postponed. It is not in the power of the reporter to present a full analysis of the contributions to be noticed; nor will any material space be taken up with comments or annotations. The province of his report, therefore, must be that of an indicator only; and with this view a mere reference, in some instances, may be made to serve the purpose of a more extended account.

*Post-Fascial Abscess, Originating in the Iliac Fossa, with a New Method of Treatment.* GURDON BUCK, M.D., Surgeon to the New York Hospital. (N. Y. Journ. of Med., March, 1857, p. 147.)—This interesting paper presents a minute history of seven cases of "a class of abscesses which," says the author, "might easily be confounded with others, the prognosis of which is less favorable, and in which the same treatment might hasten the fatal termination to which they so generally tend. The particular mode of treatment adopted in these cases, it is believed, has not hitherto been generally employed. It was first suggested by a careful study of the surgical anatomy of the parts involved; and the successful results which have followed its use may claim for it a favorable consideration."

We regret being obliged to omit the details of the cases, and to confine our quotation from the narrative to the author's description of his method of opening the abscess. It is given, in the account of the first case, as follows:—"An incision, one inch in length, was made at a finger's breadth below the middle of the outer half of Poupart's ligament through the skin, subcutaneous tissue, and fascia lata; a probe was then passed deeply upward and backward, and on being withdrawn, was followed by an escape of pus; whereupon the track of the probe was enlarged, and a free outlet afforded for the contents of the abscess, with evident relief to the patient." In another case, after the incision "down through the fascia lata," a grooved exploring needle was passed upward and backward to the depth of about two inches; the opening was then enlarged and the matter withdrawn. In a third case, the incision through a tense and unyielding fascia was followed by the introduction of a trochar exploring needle in a canula, up and backward into a free cavity, from which pus escaped through the canula. In a fourth case, after the incision and passing of the needle trochar and canula, upward and backward behind Poupart's ligament, a freer outlet was secured by the enlargement of the track, with a narrow-bladed knife passed along the side of the canula before its withdrawal, and by the subsequent temporary employment of a tent.

We append the concluding remarks without attempting to condense them.

"Of the seven cases reported above, five were males and two females. Their ages ranged from twelve to fifty-four years. In four of their number, the disease occupied the left side; and in three, the right. In one of the two female patients, the disease occurred during the puerperal state.

"*Anatomy.*—The importance of an accurate knowledge of the relative anatomy of the parts involved in this form of abscess will be admitted, if it is remembered that both the diagnosis and treatment are based upon it.

"The crural arch, which is spanned by Poupart's ligament, is divided into two halves, by a strong process of aponeurosis, sent off posteriorly from the middle of this ligament, and inserted into the ileo-pectineal eminence. The inner half serves for the passage of the great blood-vessels, lymphatics, etc.; the outer for the psoas magnus, and iliacus internus muscles, and crural nerves. The fascia iliaca, of which the above process is also a continuation, is a strong aponeurosis, which is continuous upward with the outer half of Poupart's ligament, and spreading out over the anterior face of the iliacus and psoas magnus muscles, is inserted outwardly into the inner margin of the crest of the ilium, and inwardly into the linea ileo-pectinea. It is also prolonged upward upon the psoas, as high as its origin from the lumbar vertebræ. Below Poupart's ligament, and continuous with it, the fascia lata covers the psoas and iliacus, in common with the other muscles of the thigh, and, at the inner margin of these two gives off posteriorly a septum, which is continuous above with the process of aponeurosis, already described as inserted into the ileo-pectineal eminence. Now, if an incision be made parallel with Poupart's ligament, and below its outer half, down through the fascia lata, the psoas and iliacus muscles will be exposed, and the fingers can be passed upward behind Poupart's ligament so as to arrive in the fossa, behind the iliac fascia. The same track can be followed from above downward, by incising the iliac fascia transversely, and passing the fingers downward behind it and the outer half of Poupart's ligament, till they arrive on the anterior face of the thigh. Here, then, we have a well-defined track, bounded posteriorly by a bony surface, anteriorly by a strong fascia, and shut in on either side by the insertions of the fascia into the bone. Suppuration taking place in this track could only reach the surface through the fascia, by the slow and gradual process of ulceration and absorption.

"*Pathology.*—The able author of an article in the *British and Foreign Medical Review*, (page 456, April No. for 1840,) remarks:—'An abscess in the cellular tissue of the right iliac fossa, when it does not result from a blow or external wound, from perforation of the appendix or cœcum, or from child-birth, is, we believe, in almost every instance, the consequence of disease of the bones, either of the back or pelvis. There may be cases in which an abscess forms in the iliac fossa from idiopathic inflammation of the cellular tissue, but we have never seen one.' Several of the cases narrated above prove, as we believe, beyond a doubt, that suppuration may take place in the iliac fossa behind its fascia, unconnected with caries of the lumbar vertebræ or pelvis, or morbid lesions of the cæcum or colon. There is no reason why the cachectic condition, upon which the formation of abscesses depends, may not manifest itself in this particular region as well as in any other of the body. The functions of the psoas and iliacus muscles render them liable to the injurious effects of over-straining efforts, and such efforts may be the exciting cause of suppurative inflammation in the cellular tissue surrounding these muscles, especially where a general cachectic condition coexists to favor it. Such seems to have been the fact in Case No. 4.

"*Causes.*—In Case No. 1 the irritation was propagated in the course of the absorbent vessels, which had recently been the seat of inflammation, resulting in numerous small subcutaneous abscesses along their track from the inner ankle to the groin.

"In No. 2, which was a remarkable example of cold abscess, the patient, though apparently in good health when the abscess was *opened*, exhibited, subsequently, all the signs of tubercular cachexia.

"In No. 3 a low cachectic condition, induced by copious suppuration following erysipelas of the face and scalp, coincided with the development of suppurative inflammation in the iliac fossa, without any obvious exciting cause.

"In No. 4, after violent efforts, by which the psoas and iliacus muscles were over-strained, pain and lameness first seated themselves in the iliac region; and at length, while the system was suffering under depressing influences, suppuration developed itself.

"In No. 5, the only case of an adult female, the disease originated in the puerperal state.

"In No. 6 the patient's whole aspect was expressive of a hereditary scrofulous diathesis, which had manifested itself previous to the suppuration in the iliac region, by the development of a large abscess in the axilla.

"In No. 7, the escape of a foreign body from the intestine (probably the cœcum) first excited suppurative inflammation, and its protracted sojourn in the abscess perpetuated its continuance for a long period.

"*Diagnosis.*—Other forms of abscess, besides the one in question, are found located in the iliac region, which it is important to distinguish.

"1. In this, as in other regions of the abdomen, suppuration may take place in the muscular parieties, or in the cellular tissue between the muscles and peritoneum. Such an abscess would not, however, be confined within the same precise limits; nor, in case it was seated in the cellular tissue of the fossa anterior to its fascia, could it extend behind or below Poupart's ligament. Retraction of the thigh would not be produced by it, and adhesion of the skin and subjacent tissues, as well as redness and inflammatory œdema, would take place early in its progress.

"2. Abscesses form in consequence of perforation of the vermiform process and cœcum. The suppuration in this case is commonly confined by adhesions between the peritoneal surfaces of the intestines and parieties, and the tumor produced is not confined within the same anatomical limits as the abscess in question. The same deviation from the anatomical characters already so often insisted on, is observable in abscesses produced by fecal obstruction, as well as those that are developed in the appendages of the uterus during the puerperal state.

"Ordinary psoas abscess, which is understood to depend on disease of the vertebræ, may and does, in its descent along the psoas muscle, sometimes keep behind the iliac fascia, and occupy the same track as the idiopathic abscess; but its connection with the vertebræ can be readily ascertained by careful inquiry.

"4. The question may arise, whether caries of the os ilii itself may not cause suppuration in the fossa, and present a swelling confined within the same anatomical limits. Doubtless it may, and so also may a fracture of the ilium. The author believes he has seen such an example after fracture, where, besides other abscesses around the pelvis, one formed and occupied exactly the limits of the fossa, and was evacuated by an opening made below Poupart's ligament.

"The true post-fascial abscess originating within the iliac fossa from idiopathic inflammation of the cellular tissue, may be described as follows:—It presents itself, though much more rarely, in a chronic as well as acute form, and locates itself in either the right or left iliac region. Though it may occur in early youth, it is most frequently met with in adult age, and affects alike both sexes. The tumor which it forms rises up from the hollow of the ilium, pushing before it the fascia and outer half of Poupart's ligament, so that the crest of the ilium and the anterior superior spinous process can no longer be grasped between the thumb and fingers. The outer half of Poupart's ligament is rendered tense and unyielding, and a deep-seated induration may extend two or three fingers' breadth below it. The precise limits of the swelling can only be appreciated by the touch and percussion over its abdominal portion; upon the surface the eye only perceives a fulness or a diffused swelling of these parts. No increased heat or redness of the skin existed in either of the cases above narrated. In four of them (Nos. 1, 4, 5, and 7) no fluctuation could be detected; in two (Nos. 3 and 6) it was obscure and doubtful; while in one only (No. 2, the cold abscess) it was unequivocal. In all (except No. 2) the thigh was retracted, and attempts to straighten it caused pain. In all the cases (except No. 2) constitutional disturbance and fever accompanied the local disease. In all the acute cases, the absence of heat, redness, adhesions, œdema, and also of fluctuation in three of them, is to be attributed to the remoteness of the abscess from the surface, and its confinement beneath the fascia.

"*Treatment.*—The treatment of abscesses in the iliac region, laid down by surgical authorities, does not differ materially from that of abscesses situated elsewhere. Means suited to produce resolution of the inflammation and prevent suppuration are advised in the early stage. When these have failed, such means as will hasten the approach of matter to the surface, and thus afford an opportunity to evacuate it by an opening, are then to be resorted to. In this form of abscess, the first object would be very rarely attained, owing to the remoteness of the seat of inflammation from the surface, and its confinement beneath a strong and tense fascia. For the same reason, the approach of suppuration to the surface would be very tardy, and hence great danger might be apprehended from the injurious effects of long pent-up matter, such as caries of the os ilii, as was threatened in Case No. 3, where denuded bone was encountered at the bottom of the abscess; also, deep burrowing along the walls of the pelvic cavity and openings into the peritoneum, intestines, vagina, and bladder. With the view of averting these disastrous consequences, a point was sought where an opening might be early established for the escape of the suppuration. The point selected possesses the advantage of safety in respect to its anatomical relations, it being remote from the peritoneum and important blood-vessels; it affords, also, the most dependent outlet. The mode of performing the operation has been sufficiently described in the preceding narrative. No time should be lost in resorting to it; the absence of fluctuation need not deter from it. The phlegmonous character of the swelling; its anatomical relations to the iliac fossa and Poupart's ligament; the absence of disease of the lumbar vertebræ, and the coexisting retraction of the thigh; these points being clearly made out, are sufficient to warrant the conclusion that suppuration has taken place in the fossa behind the fascia.

"It is hardly necessary to add that the approved auxiliary means of abating inflammation, such as leeches, poultices, etc., together with appropriate constitutional remedies, are not to be omitted."—pp. 159–163.

*Abscess of Posterior Chamber of Fascia Lumbarum.* M. GUNN, M. D., Prof. of Surgery in the University of Michigan. (Medical Independent, Detroit, Oct. 1857, p. 469.)—After a description of the anatomical peculiarities of the posterior abdominal fascia, Dr. Gunn continues:—

"An abscess occurring within this sheath, will naturally present peculiarities, occasioned by the shape of the chamber, distance from the surface, and the unyielding nature of the tissue which forms its walls. These peculiarities consist mainly in the absence of those physical signs which usually indicate an abscess. Bound down by the posterior layer of the fascia, there is but little swelling—a mere fulness of the region, which, perhaps, can only be seen by comparing the two sides. Fluctuation will be very obscure in the early stages, from the depth and small amount of pus; and in the later stages, from over-distension of the sheath which constitutes the walls of the abscess. Diagnosis must be based, first, upon those constitutional symptoms which indicate the suppurative process; and second, upon physical signs, modified as above described."

Three cases, of some six weeks, seven weeks, and some months in duration, are reported, which terminated happily after the evacuation of the purulent cavities, through deep incisions in the lumbar region, about two inches from the spine and on a level with the last rib.

*A New Tourniquet.* S. D. Gross, M.D., Prof., &c. (North Am. Med.-Chir. Rev., Jan. 1857, p. 96.)—This instrument is a forceps-shaped compressor, resembling somewhat a pair of double calipers, opening on a central screw-pivot, with long blades of large curvature at one end and short blades of small curvature at the other, both pairs of blades being regulated by the same rachet. A double tourniquet or a pair of tourniquets is thus produced,—a large one for the thigh and a small one for the arm or for the thigh of a small person. The upper and lower blades on each side of the central screw differ also in length and curvature. The upper blades are shorter, narrower, and of a smaller curve; their extremities, being each provided with a pad which works upon a screw, dip downward so as to direct the pad upon the artery, and by means of a few turns of the screw of the pad, to press the latter firmly on the vessel. The lower blades are longer, broader, and more widely curved, being intended to maintain the counter-pressure on the outer side of the limb which the instrument is intended to grasp, while avoiding circular constriction, as in the tourniquet of Dorsey and the later instruments of Signoroni, Skey, and others.

For a better comprehension of the instrument we must refer to the wood-cut illustration which accompanies the inventor's description.

The advantages claimed for it are—first, facility of application; secondly, the amount of pressure commanded; thirdly, its ready adaptation to limbs of different sizes; fourthly, its confining the pressure and counter-pressure to the two opposite surfaces where alone it is wanted; and lastly, the ease with which it may be slackened or removed at any stage of the operation. With a slight modification it might readily be adapted to the femoral artery under Poupart's ligament, or to the external iliac just above, or to the axillary artery.

*On the Ligation of Arteries.* T. P. Gibbons, M.D. (North Am. Med.-Chir. Rev., Jan. 1857, p. 78.)—The object of the method proposed in this paper is to lessen the danger of secondary hemorrhage "consequent upon the application of the ligature immediately below a large collateral branch."

The peculiar plan of an operation, in which the ligature was applied midway between two collateral branches, is thus described:—"The artery was exposed in its sheath to the extent of half an inch; a grooved director was carried *obliquely* under it, and raised so as to allow the ends of the instrument to rest upon the edges of the wound; the director was then brought round at right angles to the course of the vessel, and an eyed probe, armed with a ligature, carried along the groove. The artery was tied at this point."

"The mechanism of the process is simple. When the director is carried obliquely under the vessel, as the primary step, and subsequently moved round at right angles with it, it will be observed that the position which the instrument holds beneath the artery is such as to insure the application of the ligature midway between the two nearest points of resistance; which points usually to the connection of the collateral branches. The reason for its occupying this particular position is sufficiently evident; in moving it from a branch the resistance becomes less, while in moving it toward one, the resistance is of course increased; the consequence is, that the director, when placed at right angles with and under the vessel, naturally assumes a position where the two forces act equally; that is, equidistant from the two points of resistance."

*Early History of Ligature of the Common Carotid Artery, with a Report of the Unpublished Operations in the City of New York.* JAMES R. WOOD, M.D., Surgeon to Bellevue Hospital, &c. (New York Jour. of Med. for July, 1857, p. 9.)—This paper is the result of a desire in its author to place on record his own experience in this operation, and to obtain, also for publication, that of the other surgeons of New York, as shown in the reports of their numerous unpublished cases; so as to present "all the attainable unpublished material of our city bearing on this operation."

After a brief sketch of the early history of ligature of the common carotid artery, in which due honor is done to the first American cases, he gives in detail nine cases of his own. These are followed by *remarks on forty-four cases of ligature of the common carotid artery*, communicated by Prof. Valentine Mott, from which we present the following extracts:—

"In the five instances in which I have tied both carotids in the same patient there has not been any secondary hemorrhage. The interval between the operations was from two months to one year. One exception to this must be made, in which, with the consent of the patient and the approbation of my colleagues, I tied the second carotid after an interval of about fifteen minutes; the case was altogether desperate and one of great suffering. A few hours after the operation, coma came on, followed by stupor, which ended in death within forty-eight hours from the operation.

"In two cases I have tied this artery at a very early age. Both were for aneurisms from anastomosis; one was in the orbit, and had passed over the bridge of the nose into the other orbit; the other was three months old, and the tumor was of an enormous size, upon the upper part of the neck, involving the angle of the jaw and temple. They both recovered.

"In two instances I have tied it upon the distal principle for aneurism of the innominata. The first was upon a counsellor-at-law in this city, about sixty years old, of a vitiated habit. About the period of the separation of the ligature, slight arterial hemorrhage showed itself, and, shortly after, the ligature came away spontaneously. A more profuse bleeding took place from time to time, and he sunk under it two or three days afterwards.

"The other case was upon a farmer from New Jersey, forty to fifty years of age, of apparently good health; the aneurismal tumor was nearly the size of my fist, with the blood all fluid.

"In this, as in the former patient, there was scarcely a discoverable pulsation in the axillary artery, none in the brachial, nor any in the radial or ulnar.

"The common carotid was, therefore, tied on the distal side of the aneurism; everything went on favorably, the ligature separated on the thirteenth day, the wound healed kindly. In six weeks he returned home, without a vestige of the tumor remaining above the sternum.

"There was no return of the aneurismal tumor above the sternum. He died about twelve months after his return home. The autopsy revealed the existence of the aneurism in the arteria innominata, which had shriveled and contracted to a hard and very compact spherical mass.

"As before stated, one of my patients died from secondary hemorrhage, a day or two after the separation of the ligature; two died before the ligatures were cast off. One of these was where the second carotid was tied after an interval of fifteen minutes; the other was the monstrous case of osteo-sarcoma of the lower jaw, (Prince's case, see *Journal of Medical Sciences,*) in which the tumor was nearly the size of his head; the bone was sawed through at the first bicuspid tooth; and the jaw removed at the temporo-maxillary articulation of the opposite side.

"He sustained the operation remarkably well, but appeared to die from the collapse which followed it on the third day.

"In all my operations upon this artery, which now amount to forty-four, only one ligature, a small and round one, has been applied to each vessel.

"*Conclusions.*—The conclusions to which I have come are the following:—

"That in malignant disease of the nares, antrum, sides of the head, posterior fauces, and orbit, ligature of the common carotid of the side affected is not only a safe, but proper operation. If the disease is not arrested by the tying of one carotid, the other ought also to be tied, as soon as the increase of the disease is in the slightest degree manifested.

"In several of each of these classes of cases I have operated myself, and have seen it done by others, and never without manifest advantages to the patient, provided a recovery from the operation has followed. It is well known that some have only lived three to five days after tying the first carotid.

"I have seen a case lately, a malignant tumor in the posterior fauces, originating, probably, from the periosteum and bodies of two or more of the cervical vertebræ, closing one side of the posterior nares, obliterating the Eustachian tubes, and impeding deglutition, which was greatly benefited by tying the carotid of that side. The tumor obviously diminished in size, and all the unpleasant symptoms were assuaged.

"When he left for home, he promised to return and have the artery on the other side tied, as soon as there was a return of his suffering.

"In the first case of this frightful affection in which the artery was tied, the tumor actually sloughed.

"In four instances of this disease, which we had previously met with, and in which the artery was not tied, they all lingered out a most painful and distressing existence.

"I have seen and known more than one year elapse before it was deemed necessary to tie the second artery. During all this time, the disease was not arrested, but atrophy was constantly going on, and upon tying this second artery, the tumor, *though malignant,* has entirely disappeared.

"Two instances of this kind I can now refer to, in which the individuals have enjoyed good health for years without a vestige of the disease remaining.

"In idiopathic epilepsy, or, that arising from a cerebral cause, I have seen only temporary benefit from tying one carotid. In one desperate case I tied the second carotid within six months of the first, with only a mitigation of the violence and frequency of the fits. He died in less than twelve months after the last operation of tubercular phthisis.

"In six cases in which I tied the common carotid, before removing large portions of the lower jaw, for huge osteo-sarcomatous disease, and three at the temporo-maxillary articulation, I have been pleased with this preliminary step. Not only is the loss of blood comparatively trifling, but the inflammatory tumefaction of the posterior fauces is decidedly less.

"When I began these operations, now thirty-six years since, I had no guide; there was no precedent that ever came under my notice; they were original with me; my object then was the safety of my patient and the success of the operation, both of which followed in a number of my early cases.

"This step I have not deemed necessary of latter years, and would not now recommend in practice. But at that period of operative surgery I deemed it prudent and proper, and although this has been, perhaps, treated unkindly by some, they could and would not have done better than myself."—pp. 36–39.

Then come reports, from different surgeons, of some thirty-nine interesting cases, the details of many of which are given in full. We have only room for Dr. Wood's summary.

"The common carotid was ligatured for the following causes:—*Hemorrhage.*—Whole number, nine; of which six recovered and three died. *Cause of Death.*—Two became hemiplegiac; one, a few hours after the operation, dying comatose, no autopsy; the other on the second day, also dying comatose, and revealing, on *post-mortem* examination, softening of the brain and inflammation of the pleura. One died on the eleventh day, and, on examination, was found to have had pericarditis, with collections of pus in apex of lung and liver. *Malignant Disease of Head or Face.*—Whole number, seventeen, of which four resulted in the apparent cures of the original disease; ten were decidedly benefited, growth of tumor being for a time arrested; two died; one not noted. *Cause of Death.*—In one, hemiplegia supervened twenty-four hours after operation, and death occurred in sixty hours; autopsy revealed extensive softening of the brain; in the second case, death occurred three or four days after operation, from exhaustion. *Aneurism by Anastomosis.*—Whole number, ten, of which four were cured; one died; five were benefited. *Cause of Death.*—In the fatal case, phlebitis was found to have existed, pus found in cavity of deep jugular vein. *Aneurism of Branches of Carotid.*—Whole number, four; all recovered. *Epilepsy.*—Whole number, two; both benefited, but not cured. *Removal of Tumor.*—Whole number, seven; all recovered. *Secondary Hemorrhage* occurred in five instances, slightly in two, and severely in three; all recovered, the hemorrhage being controlled by pressure. *Date of Separation of Ligature* was noted in twenty-four cases,—maximum period, thirty-one days; minimum, nine days; average, fourteen days and twenty-one twenty-fourths."

*Ligature of the Primitive Carotid Artery for Malignant Disease.* GEORGE C. BLACKMAN, M.D., Prof. of Surgery, &c. (Western Lancet for Sept. 1857, p. 627.)—Dr. Blackman refers to seventeen cases, reported in the preceding paper of Dr. Wood, in which this operation was performed for malignant disease of the head and face, and recommends the operation as decidedly advantageous, according to his own experience.

These views are illustrated by the history of four cases, the first of which was published in the *Am. Jour. of Medical Sciences* for October, 1845. The effect upon the tumor in this instance was remarkable; but the patient died on the eighth day after the operation.

In the second case, a farmer's son, aged eighteen, the right primitive carotid was ligated. The ligature came away on the thirteenth day, and the whole wound was soon healed. The immediate effect, in arresting the growth of the diseased mass and in diminishing the tumefaction of the cheek, was most decided. A fungous growth soon appeared beneath the upper lip and rapidly increased. To arrest the growth of this, and to render the decay of the mass within the maxillary sinus more certain, the left primitive artery was tied on the twenty-first day after the former operation.

When the first knot was made, vision in the left eye was immediately lost. Some minutes elapsed before he ventured to apply the second; but as the sight became partially restored, the operation was completed. Not an unpleasant symptom was observed. Ligatures came away on the fourteenth day. In less than a week from the last operation the protruding growths had sloughed and separated. When last seen by Dr. Blackman, seven years after the operation, this patient was relieved of his disease and enjoying perfect health.

The third case was one of a cachectic lad, aged fourteen, very liable to excessive hemorrhage; with great tumefaction of the cheeks, right eye-ball protruding, and the right nares almost plugged up.

The primitive carotid artery was tied. Vomiting for awhile after the operation; some fulness of the head; some little fever; hemiplegia. Ligature came away on the eleventh day. In about four weeks the lad left the hospital very much improved; has not been heard from since July, 1857.

Case fourth was one of malignant disease of one-half of the brain, in a boy of seven years. There was a pulsating tumor protruding through the right side of the frontal bone, and the aneurismal bruit was unusually distinct. The first indication of the disease appeared about eighteen months before, and for six months the enlargement of the skull had been gradual and unaccompanied by pain. In August, 1856, he had a chill and convulsions which lasted an hour and a half. Next day he had five, very violent. From this date the tumor increased rapidly and the frontal pain was excruciating. Convulsions became frequent and threatening to life. Ligature of the common carotid was resorted to as offering the only chance for the patient. Convulsions set in again in twenty-four hours, and death followed in thirty-six hours.

*Femoral Aneurism cured by Veratrum Viride, Manipulation, and Compression.* GEO. C. BLACKMAN, M.D., Professor of Surgery, etc. etc. (Western Lancet, June, 1857, p. 407.)

"John Austin, aged twenty-eight, a native of England, entered the Commercial Hospital on the 7th of April. Four months previously, he felt a sharp pain along the course of the femoral artery, at the junction of the lower and middle third of the thigh, and for the first time he observed a pulsation in this region. He had worked for many years as a file-cutter, and had been accustomed to use a small anvil, which was held between his thighs. A swelling was soon detected, and this continued to increase until the time of his admission. There was a space of about three inches between the upper margin of the tumor and Poupart's ligament, and measured along the axis of the limb, the swelling was five inches at its base. The aneurismal bruit was very distinct, and the pulsations perceptible across the amphitheatre. Compression at the groin caused the tumor to diminish considerably in size, and it would immediately regain its former dimensions when the pressure was removed. The patient complained of numbness and other painful sensations in the knee, leg, and foot. As the tumor was daily increasing, and as there was no other indication of disease of the arterial system, I determined to bring the patient under the influence of veratrum viride, in order to subdue the force of the circulation. From the time of his admission he was kept on a low diet, and cathartics were administered. On the 11th I ordered six drops of the tincture every three hours. On the morning of the 12th I found that the pulse had been reduced in frequency from 94 to 65. At ten o'clock A.M. of this day he was brought before the class, when with my thumb I pressed forcibly into the aneurismal sac, for the purpose of dislodging a portion of its fibrinous layers, hoping thus partially to obstruct the artery, and to favor the further deposition of fibrin in the sac. Skey's tourniquet was now applied with moderate force between the tumor and Poupart's ligament. The progress of the case may be learned from the following record, kept by Dr. N. J. Sawyier, the house-surgeon:—

"'At 12 A.M., his pulse being 110, full, strong, and bounding, he was bled ℥ix. Pulse came down to 50, soft and regular, and continued low for several days. (The following are extracts from the Case-Book:)

"'April 13th, A.M. Suffers no pain nor uneasiness at all; slept well last night. Entire limb diminishing rapidly in size. Kept the apparatus tight. General health good; whenever any untoward symptom arose, it was promptly met, and the patient kept in a good condition. At intervals the shooting pain was felt in the tumor, but it gradually subsided altogether.

"'April 17th. Professor Blackman ordered the tourniquet to be taken off, the bandage re-applied from the toes up over the tumor, upon which it was to be tightly wrapped, and the patient to be bled, after which the following was administered:—

℞.—Antimon. and Potass. Tart. gr. $\frac{1}{4}$,

Pulv. Opii. - - - - - - gr. $\frac{1}{8}$,

Sig. Take every three hours. Patient's pulse came down to 65, soft and regular.

"'April 19th. *Souffle* ceased entirely, but the pulsation continues, though it is very weak.

"'April 22d. Pulsation in tumor has ceased altogether.

"'April 25th. Is in fine spirits; has no pain, and wants to walk about. General health very good.

"'April 30th. Has walked some steps, and complains of nothing but weakness.

"'May 21st. The pulsation in the tumor has never returned. The femoral is firmly plugged as far as the origin of the profunda, and in the popliteal space the pulsation of the artery is hardly perceptible. The tumor is daily decreasing in size, and the patient is anxious to leave the hospital and resume his business.'

"We need not add that to Mr. Fergusson, of King's College Hospital, London, is due the credit of suggesting and practicing manipulation in the treatment of aneurism, more especially at the root of the neck, where the ligature could not be applied. The combination of treatment adopted in our case, we believe, has never before been attempted, and although its value cannot be established by a single instance, we predict that it will meet with the approbation of all who are acquainted with the pathology of this affection."

*Popliteal Aneurism cured by Compression.* Drs. F. MITCHELL and JOHN W. BOND, of Mansfield, Ohio. (Trans. of the Ohio Medical Society, June 3, 1856.)—J. P., aged twenty-eight, presented a tumor of one year's growth, in the left ham, about four inches in diameter, very nearly spherical, and pulsating over its entire surface simultaneously with the arterial pulse. Compression was commenced July 25th, 1854. The foot and leg were bandaged up to the tumor; one clamp tourniquet and one ring tourniquet were then applied over the femoral; these were alternately relaxed and tightened, and their position shifted along the course of the artery. In a short time, however, another clamp was substituted for the ring. The patient, an intelligent man, was allowed to regulate for himself, in a great degree, the amount of pressure. For the first ten days compression was applied from twelve to fourteen hours in the twenty-four.

Some pain was felt during this time, but not enough to disturb the patient. His sleep was sound and refreshing; the tourniquet, however, being always first removed. The tumor had now become decidedly firmer, smaller, and the sac could no longer be emptied; œdema also was fast disappearing. For the next three weeks pressure was applied from sixteen to eighteen hours per diem; the sac becoming gradually firmer and pulsation less distinct.

Two small arteries, running longitudinally over the back of the tumor, could now be easily detected. For the next six days pressure was continued about twenty hours in the twenty-four, and for the last five days it was applied continuously. At this time all pulsation in the sac suddenly ceased; diminution in size continuing to take place. Compression was now continued for a few days longer, when exercise was allowed him, the collateral vessels becoming, at the same time, enlarged; six weeks, precisely, being occupied from the time pressure was begun until all pulsation ceased. At no time was the circulation through the artery entirely checked. The pain was never severe; the pressure along the course of the vessel being so alternated as to secure to the patient the greatest amount of ease possible. No redness or excoriation of skin at any time existed. Two years have elapsed since his dismissal, and he contiuues perfectly restored.

*Case of Popliteal Aneurism treated by Compression.* T. P. GIBBONS, M.D., of Philadelphia. (N. A. Med.-Chir. Rev., March, 1857, p. 281.)—The aneurism, in this case, was of the size of an orange, of only two weeks' standing, and in a man injured in health by intemperance and "Chagres Fever." It was painful, and accompanied by a swollen limb. Ossification of the radial artery and dilatation of the heart were discovered; and compression was therefore determined on in preference to ligature.

The limb was bandaged from the toes upward, a warm anodyne application made to the part, and compression begun on March 1st, about three weeks after the first appearance of the tumor. Skey's tourniquet was applied to the femoral artery at the inferior angle of Scarpa's triangle, pressure being changed to different points, according to the tolerance. The pressure was continued, at two or three hours' intervals, for twenty-six hours, when the instrument was removed on account of the great tumefaction of the limb. The pain, already severe, became excruciating after the removal of the instrument. Four grains of morphia were given, with only partial relief. On the third day delirium tremens supervened, and put a temporary stop to the compression. Treated at first wiih brandy, and subsequently with wine of opium enemata, and carbonate of ammonia, with porter as a diet-drink.

On the sixth day, at one o'clock P.M., the limb was bandaged, and a new instrument was adjusted. This instrument is thus described:—

"A piece of sheet-iron, curved to fit the posterior surface of the thigh, and well cushioned, forms the base of the instrument. To this three clamp tourniquets are attached by their inferior extremities. The shaft of each tourniquet is provided with a slide, so that it can be lengthened or shortened at pleasure, and fixed at any point by means of a thumb-screw. When the instrument is in place, the pads of the three tourniquets rest immediately over the femoral artery, and by turning the screw of any one of them, the vessel is compressed against the femur. But one tourniquet is intended to be used at a time, and when the pressure exerted by this becomes too painful to be borne, either one of the others may be screwed down before relaxing the one last employed. The tourniquet pads are purposely made small, to avoid pressure upon the femoral vein, if possible."

We cannot pursue the case in detail. In the course of five days the patient became able to wear the bandage constantly, and to support the compression from four to eight hours a day. The tumor then began to harden, the pulsation to lessen in force, and the general health to improve. From this time the tumor diminished from day to day, although the pressure was not kept up longer than eight hours out of the twenty-four; and upon some days was not submitted to at all.

After a slight relapse, owing to his wilful neglect of treatment, and indulgence in exercise about the house for a week, he was prevailed upon to go to bed again and reapply the compressor. It was then worn steadily for seventy-two hours; at the end of which time, upon removing it, the pulsation was found to have entirely ceased, and did not reappear. In two weeks from this date he was able to walk, with a crutch and stick, about a quarter of a mile, to his place of business, and to occupy himself at his employment, which allowed him to sit with his limb elevated. Improvement gradually went on until, with the exception of a little thickening of the tissues within the popliteal space, not the slightest trace of the original tumor could be detected.

*Traumatic Femoral Aneurism. Ligature of External Iliac Artery. Fatal Result.* FRANK A. HAMILTON, M.D., Buffalo. (Buffalo Medical Journ. for Jan. 1857, p. 466.)—A lad, aged fourteen, accidentally stabbed himself with the large blade of a pocket-knife, in his left thigh, penetrating to the depth of about an inch and a half, at a point one inch below Poupart's ligament, and over the course of the femoral artery. Profuse hemorrhage immediately occurred, but ceased in two minutes, almost as suddenly as it had commenced. The boy fainted at once, and did not revive for half an hour. There was no return of the bleeding; nor was there any pulsation until some hours had elapsed, it being first noticed "on the second, third, perhaps as early as sometime during the first day."

"This occurred on Monday, July 28th. Tuesday a chair was placed by the side of his bed, and he was helped into it while his bed was being made up. The same was done, probably, on Wednesday. Thursday, with the aid of crutches, he walked into the adjoining room. Friday, Saturday, and Sunday he walked about these two rooms. Monday he walked to the horse-barn. Tuesday and Wednesday he was more quiet.

"I am not informed that he was seen by any physician during all this time, except as mentioned on the first day. The distance at which Dr. Saunders lived, and the absence of any lively apprehension of danger, led to this neglect; yet it is not supposed that any more active interference, even at this early period, could have prevented the final result. It is remarkable, however, considering that the artery had been completely severed, and considering that the wound of the skin, half an inch in breadth, had so recently closed—it is certainly remarkable that with all these circumstances existing, and with his daily exercise, it did not give way, and in a moment terminate his life. But this is scarcely less remarkable than that it should ever have ceased to bleed spontaneously, or with such apparently insufficient means [pressure with the fingers, cold water, and rest on his back,] as were at first employed by the terrified assistants.

"On Wednesday, August 6th, ten days after the accident, a compress was, for the first time, applied. On Thursday, a messenger having been sent to me for advice, Charrière's compressor was substituted, but proving very painful, the simple compress, with a bandage, was, after a few hours, again employed; and this was continued until Saturday, August 9th, the thirteenth day after the accident, when I first saw him. He was at this time exceedingly pale, with a soft, feeble pulse. The aneurism, then of about the size of a hickory-nut, could be distinctly seen pulsating as he lay upon his back. Pressing my finger upon it, the blood seemed to be near the surface. Continuing the pressure, the sac became emptied, and on removal of the finger it was not immediately refilled. The aneurismal thrill, or vibration, was very intense in the tumor, and continued downward, along the line of the artery, about four inches; and upward as far as the pulsations of the artery could be felt underneath Poupart's ligament.

"I determined, at once, to make another attempt to close the aneurism by pressure; and as the space above, to which the instrument could be effectually applied, was only about one inch, and as it was evident that the aneurism could be emptied by direct pressure, I concluded, at first, to place the pad of the instrument over the aneurism and not above it. Accordingly, this was done, and I left him again in charge of Dr. Saunders.

"A few days after it was found that the cicatrix, over the aneurism, was beginning to ulcerate, from the constant pressure; and as no improvement had taken place in the condition of the aneurism, at my request the instrument was removed to a point directly over the pubis. Soon the pain from the pressure here became insupportable, and, at my instance again, on the 30th of August, leaden weights were substituted for the compressor.

"The directions were always that the pressure should be such as to diminish the current of blood in the artery, but not to arrest it. They were to apply sufficient to prevent the 'thrill.' The direction and exact point of pressure were also to be as much varied as the circumstances of the case would permit.

"The leaden weights were made fast in a belt, which was secured around the hips. They were conical, and varied in size and in weight. During most of the time he was able to sustain seven and a half pounds without much inconvenience. Indeed, this was found to be a much less painful mode of pressure than the compressor, even when the amount of pressure, as determined by its effect upon the aneurism, was precisely the same. He was especially relieved by the removal of the pressure made by the posterior pad of the compressor. He was able to turn partly upon either side while wearing the weights.

"Sept. 19th. After six weeks of continued pressure, I found the aneurismal tumor and the thrill as when I first saw it. The lad was becoming gradually more feeble, and his left limb œdematous. It was apparent that pressure could not accomplish the cure; accordingly, assisted by Dr. Saunders and his brother, with Messrs. Mason and Flint, I proceeded to tie the external iliac artery. We gave the patient chloroform; and having opened underneath the peritoneum, by a long incision above and parallel to Poupart's ligament, I exposed the artery one inch above the root of the internal epigastric artery, and secured it with a strong ligature. The pulsation in the artery below immediately ceased.

"When the effects of the chloroform had disappeared, George began first to complain that he had no leg, that we had cut it off, etc., indicating that he had no sensibility in the limb. Soon he said it felt like a log, it was so heavy; and within half an hour he began to speak of pain in his knee and in his shins, etc. From this time forward these pains became more and more intense, until the limb was amputated.

"In a few days white spots were seen on his shins, and his toes began to grow purple and black. The blackness gradually extended up the limb—the most part of his foot becoming dry and shrunken. The death gradually extended to the knee. As early as the fifth day after the ligature was applied, a slight thrill was discovered in the aneurism again. and this continued without change until the time of death. There was, however, no pulsation.

"On the 16th of October I amputated his thigh a little above its middle. The bleeding was inconsiderable, and the flaps looked tolerably well. He submitted to this operation, also, while under the influence of chloroform, and afterward rallied as before. Subsequently, however, on the second or third day, he began to sink, and died on the fifth day, and thus at last he was released from his sufferings.

"From the time of the receipt of the injury he lived twelve weeks and one day."

The report of this interesting case is completed with a wood-cut, representing the wounded artery, with its aneurismal sac and adjacent vein, as prepared by Dr. Charles Ap A. Bowen, of Buffalo, who furnished the drawing, and accompanied it with a detailed description of the parts, as carefully dissected by himself.

*Case of Ligature of the Common Iliac Artery, for Aneurism of the External Iliac. Death.* W. H. VAN BUREN, M.D., Prof. Anat. University of N. Y., Surgeon of N. Y. Hospital, etc. etc. (N. Y. Journ. of Med., Jan. 1857, p. 57.)—The subject of this case, a professional gentleman, aged forty-six, unmarried, of irregular, intemperate habits, and obese in person, first consulted Dr. V. B. in May, 1853. He had noticed a tumor, then about the size of a hickory-nut, in the fold of the groin, over the course of the main artery, immediately below Poupart's ligament. The disease appeared to be spontaneous in origin.

"The tumor was now about the size of an ordinary child's head at birth, somewhat flattened upon its surface, occupying the fold of the groin, and extending upward along the course of the external iliac artery, beneath Poupart's ligament, the position of which was marked by a depressed line dividing the convex surface of the tumor into two equal halves. Its bulk was diminished very considerably by pressing the external iliac artery against the brim of the pelvis, and its pulsations were entirely checked by the pressure. The patient's abdomen was very fat, and continued pressure caused so much pain that I could not satisfy myself that the contents of the tumor were entirely fluid; but, from its rapid diminution, and also from its well-marked expansive pulsations, I concluded that it contained but a small proportion of coagulum.

"He was urged to submit to an operation, but hesitated and procrastinated until the latter part of November, six months longer, during which time the tumor had considerably more than doubled in dimensions. His aneurism had increased to a fearful size; it was still pulsating furiously, and its contents, as far as I could judge, were yet mainly fluid. Upon the most prominent part of its surface (above Poupart's ligament, where the great bulk of the tumor now lay) was a dark-colored eschar, larger than a half-dollar, and the remainder of its surface presented a dusky, livid hue. The limb below was swelled to double its natural size. His pain was constant, and, at times, very severe."

The ligature of the main arterial trunk supplying the tumor was determined on, and performed while the patient was under the influence of chloroform. The preliminary steps of the operation were somewhat embarrassed by the corpulence of the patient, but the vessel was soon reached, and carefully exposed for the space of three-fourths of an inch; and being recognized to be healthy, a ligature was readily thrown around it and tied, just half an inch above the bifurcation into internal and external iliacs, with the result of at once arresting all pulsation in the tumor.

The operation, on the whole, was well borne; the shock was slight, and reaction immediate and favorable. The temperature of the limb was, for a few hours, lower than that of the other, but afterward did not vary from that of the rest of the body. The patient did well until the close of the second day, when he had a chill, succeeded by fever; and the tumor, then reduced to one-third of its former size, became exceedingly tender and painful. These symptoms continued to increase, and the strength to decline, until death ensued at the close of the fourth day.

Post-mortem examination the next day.—The tumor and surrounding parts were found to be the seat of inflammation, which had resulted in suppuration. It contained recent coagula, with some layers, slight in thickness, of older date, and involved the external iliac to within an inch of its bifurcation. Wound had united throughout, except in the track of the ligature. There was no peritonitis; death having resulted from the irritative fever, which accompanied the suppurative inflammation in and around the aneurismal sac. On laying open the arteries in the vicinity of the ligature, a firm, conical coagulum was found, both above and below the place of deligation; the latter clot being more than half an inch in length and bifurcated—one portion being prolonged into the internal and the other into the external iliac arteries.

*Aneurism of the Crural Artery. Ligature of External Iliac.* H. DARET, M.D.P. (N. O. Med. and Surg. Journ., vol. xiii. No. 4, p. 471.)—Felix L., of New Orleans, about eighty-six years of age, enjoying ordinarily good health, of a nervous temperament, presented an aneurism of the femoral artery, extending from the crural ring to three fingers' breadth below. Several means, and among others compression, were tried, without success.

The external iliac was deligated, June 19th, 1842, by the "process of M. Velpeau," at the passage of the artery upon the internal edge of the psoas; no anæsthetic was employed or needed, and but little blood was lost. No unusual symptom or accident occurred until on the 23d, four days after the operation, when "there passed by the course of the ligature quite a large quantity of blood." "This only ceased on the 27th, under the influence of styptics internally, and the constant application of ice to the wound." It was supposed to arise from an untied and open branch of the tegumentary artery. "From this date, the state of the patient left nothing to be desired." Ligature did not come away until forty-five days after the operation, very slight trachion only having been made, and this only in the last days of the period. Patient began to walk on the eighteenth day. There remained only a small hard knot in the place of the aneurism. His health continued good until 1846, when he began to experience, at long intervals, attacks of nervous disorder, which augmented in intensity, and assumed the form of a species of angina pectoris, and becoming more frequent toward the end of 1848, finally terminated in sudden death, about six years after the ligature of the external iliac.

*Aneurism of the Crural Artery. Ligature.* DR. MERCIER. Reported by DR. M. ESCOUBAS. (N. O. Med. and Surg. Journ., vol. xiii. No. 4, p. 472.)—Louis F., aged forty-six, of a constitution a little shattered, and of a scrofulo-nervous temperament; affected with varicose veins and fibrous rheumatism in the pelvic members, with his neck marked with mucous cicatrices of old lymphatic suppurations; presented August 26th, 1856, an aneurismal tumor as large as a full-grown fœtal head, on the superior part of the middle-third of the right thigh. It had been first perceived about fifteen months previously, being then "of the size of a small hen's-egg."

A ligature of three threads was applied by Dr. Mercier, September 2d, with a modification of Deschamp's needle, to the femoral artery, about an inch and a half below the profunda. The pulsation of the tumor was entirely checked: no blood was lost. The patient was subjected to the influence of chloroform during the performance of the operation, and bore it well. The entire member was enveloped in warm sand. The temperature of the limb was 'satisfactory' on the second day; no unfavorable symptoms presented; dressing changed on the sixth; union of edges of wound complete, except at the outlet from the ligature; the ligature came away without effort or hemorrhage on the nineteenth day. November 24th. Patient has resumed his ordinary occupation of a knife-grinder, improved in general health, and released from the weight, and pain, and other inconveniences of his aneurism, the tumor having been reduced to the size of an ordinary orange.

*Aneurism of Femoral Artery. Ligature of External Iliac.* Reported by DR. A. MERCIER. (Ibid. p. 483.)—Michael Moran, aged thirty, native of Ireland, resident fourteen years in New Orleans, of good health, and rather strong constitution; was injured about eighteen months previously, while employed in a hardware store, by letting fall upon his right groin a box, which he was attempting to raise, weighing nearly one hundred and fifty pounds. It was only some days later that he perceived that a tumor, of the size of a hazel-nut, existed in the abdomino-crural fold. The subsequent history of his case, and a very complete and lucid description of the physical characteristics observed in the tumor and the neighboring regions, are given by Dr. A. Mercier. After suffering for several months, and persevering in the use of a bandage to the limb, with occasional rest in his room and soothing applications to the tumor, he entered the Charity Hospital. Here compression with the double-cushioned tourniquet was tried, but could not be endured, on account of the agonizing pain. After remaining ten weeks in this hospital, he put himself under the care of Dr. Mercier.

"Convinced that, in the actual state of things, compression would be completely useless, and that the time was not far distant when the sanguineous stream would open an issue through the walls of the cyst, he believed that the only means of cure which remained consisted in the ligature of the external iliac. The patient upon whom it would be practiced was a young man of good constitution, an old client of Dr. Mercier, in whom he had the greatest confidence; he was firmly determined, and he demanded, with importunity, that as early a day as possible should be fixed for the operation."

It was performed on the 9th of September, the patient being under the influence of chloroform. The vessel was of the ordinary calibre and perfectly healthy; the pulsation was immediately checked; the temperature of the limb was not affected; no pain was complained of except at the incision. The only symptoms deserving attention were those of incipient peritonitis. The limb preserved its temperature and sensibility unabated, but was relieved from the pain which had affected it throughout, and especially at the knees, before the operation. The ligature came away near the end of the thirteenth day, leaving the patient in a perfectly satisfactory state of health. On the 29th, the twentieth day, he was out of bed, with the wound nearly closed; and found himself strong enough to resume his ordinary work, the wound being healed, on the first day of November, just twenty-three days after the date of the operation.

*Tetanus, after Operation for Necrosis, Cured.* DR. J. J. CHISHOLM. (Charleston Med. Journ. and Rev., vol. xii. No. 5.)—A slave, aged twenty-three, "stout and in good health," except a necrosis, two years old, of the inferior and anterior face of the femur. This anterior necrosed portion of the femur was removed May 23, 1857, with the gouge, through a free incision in front of the thigh. Four fluidounces of chloroform had to be administered before anæsthesia was induced; cold water dressing was the only application, except the pledgets of lint which were employed to prevent the wound from closing over the denuded surface.

The patient would walk about the hospital, when not closely watched, a few days after the operation; and on the fifteenth day he took a long walk under an intensely hot sun. The next morning soreness of the throat and uneasiness in swallowing appeared, and continued five days; on the sixth day Dr. C. "found the jaw very much contracted; not opened more than one-quarter of an inch; and during the visit he had a severe cramp, accompanied with opisthotonos. He complained of severe pain in the throat and difficulty of swallowing, the face presenting the peculiar and striking expression so characteristic of tetanus."

Patient to be kept quiet in a dark room; nourishment and stimulus ad libitum; thirty drops of laudanum to allay pain, and a fluidrachm of laudanum at bedtime. The patient was found to be worse on the second day of treatment; paroxysms violent and very frequent; pain in the throat and sense of suffocating, almost entirely disabling him from taking food or drink. An inhalation of chloroform was prescribed, which removed this disagreeable symptom, and enabled him, during the visit, to drink nearly one pint of porter. "Chloroform to be inhaled before taking food; nourishment and stimulus to be urged."

The further treatment was not detailed. It was based on the belief of the narrator "that tetanus destroys its victims chiefly by exhaustion; and if by the administration of stimulus and easily digested and nutritious food, the patient can be kept alive a certain number of days, the irritability of the nervous system will wear itself out, and the patient will be saved."

"The treatment, instituted and continued with very little change, consisted in inhaling chloroform when the spasms of the throat were so severe that the patient could not swallow. This inhalation was never carried to insensibility, or even to drowsiness, and was only required on three or four occasions; from thirty to sixty drops of laudanum were administered at bedtime, which generally gave him a good night's rest. After a few days the laudanum nauseated him, and he vomited frequently. This was then changed for morphine, half a grain every night at bedtime; hot poultices to the abdomen, when very hard and painful; two drops of croton oil every fourth day, to open the bowels—for they were never opened naturally for three weeks—until the paroxysms had very much mitigated; last, not least, strong plain nourishment, even to forcing; porter I considered peculiarly applicable for the anodyne effect, as well as its sustaining powers. This was used without limit. I was always most satisfied when the patient had taken his four bottles in the day, for he was sure to have a good night after such copious potations."

The paroxysms gradually lessened in severity and frequency, until they finally left him; occasionally, however, the paroxysm was a violent one during this improving period; and even a month after the outset of the attack, when he was well enough to walk about, he had one, while standing on the floor, which threw him on his back.

Dr. C. states that in the last three years he has successfully treated three cases of tetanus; and he attributes his good fortune to the course of treatment, above described, which was adopted in each case.

*A Lecture on Traumatic Tetanus, with Notes of Cases.* HENRY F. CAMPBELL, M.D., Prof. of Surg. Anat., etc. (Southern Med. and Surg. Journal, Feb. 1857, p. 75.)—We are able only to quote the conclusion of this clinical lecture, which presents a practical review of the cases:—

"You will observe that in all three of our cases, the tetanic state was preceded by a wound; that in Case 3d, the ordinary stimulus of light produced a fixed contraction in the motor muscles of the eye, causing strabismus; afterwards on turning the face to the light, twitching of the facial muscles occurred, and on touching lightly the cheek, violent jactitations in the muscles of the neck ushered in the characteristic opisthotonos; while in Case 2d, on carefully raising the affected hand to examine the cicatrix, the patient was so violently convulsed that he fell from the chair upon the ground. The presence of the urine causes spasmodic stricture at the neck of the bladder, while in all these instances the authority of the will is ignored, and a universal automatism holds empire over the muscular system. How is this brought about? you ask. The wound *probably* excites, and in some manner permanently exalts, this excito-motory function of the nerves, but in *what* way, we cannot at present safely answer.

"As to treatment, I think we may safely say that our cases seem to favor the opinion, that in the early stages of tetanus, sedatives and revulsives are beneficial, while in the latter stages, stimulants should claim the first place. Chloroform, in heavy doses, will produce, in our opinion, all the sedation we could desire from any one remedy, while in the latter stages, we can still recommend it, but in smaller doses, or in the manner in which it was applied in Case 3d; viz., in combination with another anæsthetic of a more stimulating character. We have great reliance on this combination.

"In regard to revulsives, the idea just now occurs to me, more from a consideration of the nature of the disease than legitimately from a review of the above cases, that I would not recommend you to apply them as a prominent part of the treatment, for I think they are more calculated to enhance and exalt peripheral excitability without materially improving the condition of the disordered nervous centres. Although we have generally applied them freely, I must confess that I cannot recollect, at present, any instance wherein marked benefit has acccrued from their use, and sometimes I have seen decided injury follow their application.

"There are many other ways of viewing the pathology of this disease, scattered everywhere throughout the books and throughout the journals, and also many other modes of treatment. Amputation of the entire part, or a section of the nerve, are both rational, the last especially, and should be *considered*, in every case of the disease; Indian hemp (*canabis indica*) has, it is said, done much in many cases; and Dr. R. B. Todd recommends a long bag of ice to the spine. I would probably use one or all of these remedies in certain cases, but at present, and from the few cases I have had the opportunity of comparing, I would never allow chloroform or brandy to be excluded, except under very peculiar circumstances, from the medication."

*Case of Exostosis occupying the Orbit and Nasal Cavity, successfully removed, and Vision restored.* ALEXANDER B. MOTT, M.D., etc. etc. (Am. Journ. Med. Sci., Jan. 1857, p. 35.)

In this case Dr. Mott diagnosticated exostosis occupying the left nasal cavity and orbital foramen. The extent of the growth of bone could not be felt, but, from the duration of the malady he inferred that it penetrated far into the socket, and was firmly attached to the orbital plates.

"The patient, William Hoy, aged thirty-three, a native of Edinburgh, Scotland, and a wood-turner by trade, had, about seven years previous to his applying to me for advice, noticed an enlargement toward the inner canthus of the left eye. His previous health had been good, though he had been subject to headache, and his attention was first drawn to the seat of disease by inflammation around the part, and a troublesome flow of tears over the cheek. About eighteen months after he first noticed the tumor, the left nostril was closed up, and he then applied for relief to a surgeon of Edinburgh, who passed a probe up that cavity; but the only effect of this was to excite great pain in the head and produce an extensive swelling on the side of the face. Professor Syme next examined it, but proposed no operation. At this time the tumor did not occupy the socket of the eye; but it gradually increased in size, pressing that organ to the outer canthus, and impairing its vision. Nothing, however, appears to have been done; and in this condition he progressively went on from bad to worse until, having in the interval emigrated to this country, he applied for my advice and assistance in the month of April, 1854. About two months previously, a discharge of bloody matter had taken place from the left nostril, and continued to a considerable extent night and day subsequently. The pain in the head had also become very violent, and prevented him from attending to his work. About a month after the discharge from the nostril the eyelids became swollen, and an abscess formed under the lower lid, toward the inner canthus. On passing a probe through this opening, the bone could be distinctly felt, and its existence in the nostril was also equally evident."

An operation having been determined on, it was performed on the 22d of April, in the following manner:—

"The patient, being placed on a bed, was brought under the influence of a mixture consisting of equal portions of chloroform and sulphuric ether, which, in such cases, I recommend. I then made an incision from the ala of the nose in a direct line upward to about half an inch above the superciliary ridge, and afterward a transverse one from the centre of the upper eyelid across the nasal bone to the opposite eyelid, terminating in a line with the inner canthus of the right eye. The four flaps thus made were next dissected up, beginning at the points where the incisions intersected each other, and carefully extending through all the tissues to the bone. Upon raising the flap nearest the nose, it was evident that a large portion of the osseous mass extended into the nasal cavity, and it consequently became necessary to remove the whole of the fleshy portion of the nose from the nasal bone of the left side. This being accomplished, I dissected the opposite flap clear from the tumor, keeping as close as possible to the bone in order to avoid wounding the eyeball or its surrounding tissues and their attachments. This, too, being done, I found the bony tumor was firmly impacted in the orbit and nasal cavity. I consequently separated the nasal bone of the left side from its fellow of the opposite, by means of a strong pair of Liston's bone-forceps, and with a fine straight flexible saw detached it from its frontal attachment. By a little manipulation I was thus enabled to remove the portion represented in Pl. I., Fig. 3, [*vide Am. Journ. of Med. Sciences*, Jan., 1857, p. 40]; and on accomplishing this, I next, by means of a delicate chisel and hammer, gradually detached the other bony mass represented in Pl. I., Fig. 4, [*v. ibid.*] from the orbital plate of the frontal bone, and also from the orbital plate of the superior maxillary. The os unguis was so thoroughly incorporated with the tumor that I was obliged to remove it along with the mass; and the whole being now somewhat movable, I made a slight traction by means of a pair of strong forceps. A few more cuts of the chisel enabled me to withdraw it; and, to my own great satisfaction, as well as the astonishment of all present, I discovered that the orbital plates had not been injured. Had these been so, I need not say the result would have been most serious: the brain would have been exposed, and it would have been almost impossible to have avoided a fatal issue. But this danger was happily evaded, and all now went on comparatively easy. On removal of the tumor from the orbit, a slight discharge of pus took place; accounting in some degree for the pain previously experienced in that cavity.

"The next important point which presented itself for my consideration was the restoration of the eye to its natural and original position. From attempting this I was dissuaded by some of the professional gentlemen around me, from whom I had derived valuable assistance during the operation. They recommended that the cavity should be filled in with lint; but, desirous of doing all in my power for the benefit of the patient, and seeing no reason why the eye should not be replaced—considering, also, that there was every probability of the coverings adhering, there being two raw surfaces to be brought in contact—I replaced the organ, and drew the edges of the wound together by means of interrupted sutures and adhesive straps. A bandage was next passed around the head, containing a small compress which was placed over the eyeball. The latter was thus retained in its natural cavity; my object being to fill up the orbital foramen, and have union by the first intention.

"Secondary to the recovery of the patient from so formidable and dangerous an operation, the most gratifying result in this case is the fact that the eye, which had for several years been gradually but completely pressed out of its regular position, was eventually replaced; and that the vision, which had been totally lost for several years, was perfectly restored. So soon as he was able to sit up—that is, about sixteen days after the operation—the patient called my attention to his eye, which appeared to move naturally, and to his astonishment he found that he could distinguish objects, although not very distinctly. His sight continued to improve, as well as his general health; and in about a month he was able to leave the hospital entirely cured.

"From time to time I have seen him since, and observed with pleasure the gradual improvement in his vision. In less than three months after the operation, the sight was as good in the eye operated upon as it was in the other, and his personal appearance was much improved. The eyeball had resumed its natural position, and, when I last saw him, the scar on the face was scarcely perceptible."

*Cases of Resection of the Superior and of the Inferior Maxilla.* W. H. MUSSEY, M.D., Cincinnati. (Cincinnati Med. Observer, Dec. 1857, p. 544.)—Dr. Mussey reports two cases of resection of the upper jaw-bone and one of the lower.

CASE I., April 24, 1856.—Mrs. C., aged fifty, a gradual enlargement of the left cheek, perceived during four months previously.

"The development involves the maxillary and malar bones; in the position of the latter, and below it, is a large projection with an inflamed surface, and fluctuation can be distinctly felt in the lower half of it. There is no projection into the cavity of the mouth. Believing in the malignant character of the disease, I advised the removal, as likely to prolong life; and proceeded to operate by making an incision from a point on a level with, and an inch posterior to the fronto-malar suture, terminating in the angle of the mouth; and dividing the zygoma of the temporal bone a third of an inch from the temporo-malar suture, separating the fronto-malar suture, cutting the nasal process, and detaching the maxilla from its fellow.

"The orbital ridge and floor were completely softened, and the 'fluctuation' proved to be the result of the softening of a portion of the substance of the tumor, which was encephalomatous. The disease was not confined to the maxillary and malar bones, but seemed to have invaded the deep-seated tissues posteriorly, so as to lead me to expect the return of the disease.

"The hemorrhage was abundant, though not seriously so; and the patient recovered rapidly, remaining well up to last February, since which I have heard nothing from her."

CASE II., August 19th, 1857.—Mr. S., aged fifty-one, discovered some evidence of organic disease on the left side of his mouth, about four months previously.

"At this time there is an ulcerated surface, including a portion of the covering of the hard palate, two and a half by one and a half inches. Externally there is an enlargement of the cheek, anterior to and below the malar bone.

"Regarding the affection as of a cancerous character, I proceeded to remove the superior maxilla of that side, and commenced the division of the soft parts over the zygomatic arch, terminating in the angle of the mouth. The malar bone was sawed through its centre according to the suggestion of Dr. R. D. Mussey, (*vide Transactions Am. Med. Association*, vol. iii., p. 364,) and the soft palate was separated from the border of the hard palate, leaving it otherwise intact.

"A mass of encephaloid disease, filling the antrum, had invaded the osseous structure anteriorly and posteriorly; and inferiorly and externally some of the soft parts required removal. The external wound healed readily; but internally, after four weeks, a small fungus sprouted from the inferior angle of the wound, which was subdued by the liberal application of powdered sulphate of zinc. There followed slight exfoliation of the malar bone, which was removed eight weeks after the operation."

Dr. Mussey apprehended a return of the disease, although the patient reported himself to be doing well, November 13th.

CASE III.—Miss S., aged eighteen, eight weeks previously had "felt trouble from a tooth in the lower jaw, and from that time had noticed a rapidly increasing enlargement of it."

"A tooth was extracted, but the tumor increased. I decided to remove the inferior maxilla, and operated by an incision from the middle of lip to the lower border of the jaw, and along the border to the angle; then, dissecting the flap off, I extracted the first bicuspid tooth, and sawed the bone at this point, continuing the dissection in the manner suggested by Dr. R. D. Mussey, (*Transactions Am. Med. Association*, vol. 3, p. 364,) for the preservation of the facial nerve; I disarticulated the bone without interfering with the branches of the nerve; in the progress of the operation the inferior maxillary artery was ligated before dividing it.

"The wound healed kindly, and in two weeks was closed; but three days after opened, and a fragment of the remaining portion of the bone which had exfoliated was removed, after which the wound soon closed, leaving the face symmetrical, with a very slight depression in the situation of the body of the removed bone.

"The specimen is one of osteo sarcoma."

*Resections of the Lower Jaw.* Prof. GUNN. (Medical Independent, April, 1857, p. 67.)

"CASE I.—April 13, 1851. B., aged about 25, presented himself at the college clinic, with a constantly increasing osteoma of the body of the inferior maxilla. As the tumor involved the whole thickness of the bone, resection became an operation of necessity. Chloroform was used, and anæsthesia being complete, I commenced the operation by an incision along the base of the jaw, extending from opposite the lateral incisor to the angle; another was made at right angles from the anterior extremity to the mouth. The facial artery was secured, and a flap, composed of the cheek, turned up, exposing the external surface of the jaw. The dissection was then carried behind the bone, through to the mouth at the anterior portion of the wound, a section made with Hey's saw, the bone separated from the soft parts and resected at the angle. The flap was adjusted, and the wound healed by first intention. Contraction of the internal pterygoid distorted the remaining half of the jaw, giving a wry appearance to the face, and preventing accurate meeting of the teeth. This difficulty was so great as to prevent mastication, and the patient was obliged to subsist on liquid food for many weeks, and a year passed before perfect control of the mutilated member was obtained."

"CASE II.—March 19, 1853. A gentleman, aged about 35, was suffering from a carious condition of the body of the inferior maxilla. The bone was much enlarged, and gave rise to a fœtid discharge. Resection was here, too, an operation, not of choice, but of necessity, and in the performance was almost identical with the preceding case. The closure of the wound was equally rapid and perfect. There was, however, no muscular contraction, and the patient retained full and perfect control of the remaining portion of the jaw, as evidenced by his mastication of beef-steak at the table of his hotel one week from the operation.

"In the first case, which I have repeatedly seen since the operation, a firm cartilaginoid deposit took place along the course of the removed bone. The second case I have never met since his dismissal."

*Excision of the Head and Upper Third of Humerus.* Prof. GUNN. (Ibid., p. 69.)

"CASE III.—During the winter of 1850–51, a young Irishman, aged about twenty years, presented himself at the college clinic, with caries of the head, and necrosis of the shaft of the humerus, involving the upper two-fifths of the bone. There was also partial anchylosis of the joint, caused, as was afterward shown at the operation, by adhesions in the joint. I determined on resection, and the patient being chloroformed, I carried an incision from the acromion to a point just below the deltoid insertion, through fascia and muscle down to the bone, dissected the tissues from the bone, opened the joint, my progress through which was much impeded by adhesions which nearly obliterated the synovial cavity, dislocated the head, completed the dissection, and sawed through the bone at the deltoid ridge. The circumflex arteries only required ligature, and the wound was dressed with sutures and straps. On the second day erysipelatous inflammation involved the whole arm, from shoulder to hand, which subsided with a profuse discharge of fœtid and ill-conditioned pus from the wound. Quinine, wine, and animal broth were freely exhibited, and union by first intention was secured in the upper two-thirds of the wound, with healthy discharge from the lower portion. Fourteen days from the operation the patient was discharged, and returned to his home."

"CASE IV.—Dec. 13, 1854. H., a lad fifteen years of age. Caries of the head and upper third of humerus. Freedom of motion in the joint, though the whole head seems to be in perfectly softened condition. Operation similar to preceding case, except that the main incision was met at its lower extremity by a short transverse cut. Everything progressed favorably, and on the fourteenth day the patient was discharged, and started on his return.

"These two cases are illustrative of the advantages of conservative surgery. In operating for caries we usually find ourselves in the immediate neighborhood of joints, and while we are generally called upon to sacrifice the whole joint extremity of the bone, we may by resections save a valuable member. In each of the last two cases, by the use of a firm leather splint, embracing the shoulder and arm, the patients were enabled to use the limb for most ordinary purposes. The first I have not heard from since the summer following the operation, when he was performing heavy manual labor. The lad, I heard from a few days since, and he is as useful as most lads of his age upon a farm."

Prof. GUNN reports another *Operation for Removal of the Head of the Humerus*, performed on the 5th of October, 1857. (Med. Independent, Dec. 1857, p. 576.)

"W. W. W., aged twenty-nine, was suffering from a carious condition of the head of humerus, of some months' standing. From the proximity of the joint, it was feared that the disease had extended into that cavity. The operation was, however, in its first stages, only exploratory. An incision was carried, from over the acromion, downward, dividing longitudinally the deltoid muscle, which was raised from the bone, thus exposing the diseased surface, an examination of which betrayed extensive communication with the joint. The incision was now extended, the capsular ligament divided, the head of the bone dislocated, and its surgical neck sawn through. Only one vessel required ligature. The wound was closed by seven sutures, and tepid water dressings were applied. But little febrile reaction supervened. The sutures were removed on the fourth and sixth days, when perfect union had taken place, except at a point corresponding with the fistulous opening. A generous diet, with ale or porter, supported the patient after the first three or four days from the operation. At the present date—three weeks subsequent to the operation—the wound is entirely closed, except at the original fistulous opening, which is nearly filled up. The discharge is but trivial, and the patient is attending to his business.

"It will be noticed that in this case, as in one of the two previously reported, a single straight incision only was used. This form of incision is abundantly ample, and far preferable to the flap or angular form, as it exposes less surface, and leaves less work for nature to accomplish in the healing process."

*Exsection of the Head of the Humerus.* GEORGE C. BLACKMAN, M.D., Professor of Surgery, &c. (Western Lancet, Aug. 1857, p. 551.)—The following case is reported by Dr. Wm. Hays, who, as resident surgeon, had charge of the after treatment.

"Richard Pierce, a lad aged five years, was admitted into the Commercial Hospital, April 17th. His mother states that some months previous to his admittance, while playing with his brother, he fell, striking his right shoulder against a rock. No particular notice was taken of the accident, until three weeks afterwards, when tumefaction began in the axilla, attended with considerable pain. Emollients were applied, but the tumefaction increased, and in about three months after its first appearance the abscess opened, with a discharge of a sero-purulent character.

"At the time of his admission, the lad was in pretty good health, but not as good as it was previous to the injury. There was considerable tumefaction in the axilla as well as in the deltoid region; a fistulous opening connecting the axilla with the head of the humerus and glenoid cavity, from which there was a constant discharge. The mobility of the limb was very slight, the patient being unable to abduct the elbow, or to lift the hand to the head.

"The pain being so great, the discharge so excessive, and the lad's general health seeming to fail, on the 6th of June Prof. Blackman decided to operate.

"The patient having been brought under the influence of chloroform, the head of the bone was exposed by making a triangular flap which detached the deltoid muscle. It proved to be in a carious condition as well as the upper extremity of the shaft, about an inch of the latter requiring removal, which was easily effected by means of a small Luer's saw. The glenoid cavity was also involved, and a considerable portion of it was removed with the 'bone-gnawing' forceps, which Prof. B. regards as a very valuable instrument in most operations, for detaching small portions of dead bone.

"The wound was covered with cold water dressing, and Fox's apparatus for fracture of the clavicle applied. The wound healed kindly, and on the fourth day after the operation the stitches were removed.

"Aug. 10th. The wound has healed without an untoward symptom. The lad is in good health, and has pretty good use of the limb, being able to abduct the elbow or lift the hand to his head.

"He is ready for his discharge, and there can be no question that in the course of a few months he will have nearly the perfect use of his arm."

*Excision of the Os Calcis; Improved Method of Operating, and Rapid Recovery.* CLIFFORD MORROGH, M.D., of New Brunswick, N. J. (Med. and Surg. Reporter, June, 1857, p. 284.)—This operation was resorted to in a case of destructive inflammation of the os calcis and neighboring soft parts, seriously compromising the general health, in a patient aged twelve years, who, nine months previously, had had his foot slightly squeezed between two railroad cars.

"The boy was placed on his left side on a table, and rendered insensible with chloroform.

"A vertical incision was made over the posterior extremity of the os calcis, extending from the superior to the inferior surface. This was continued along the inferior surface of the bone to its articulation with the cuboid, taking care to keep outside of the external plantar artery.

"The incision was then carried upward to a short distance above the superior surface of the bone, without wounding the peroneal tendons. This incision described a square flap on the outside of the foot, which was dissected up, and the tendo-Achillis separated close to its attachment.

"A strong narrow scalpel was then introduced under the peroneal tendons, and made to separate the calcaneo-cuboid articulation, without severing the tendons or the artery beneath.

"The knife was then introduced between the upper surface of the bone and the astragalus, made to cut the interosseous ligament, and gradually separate the articular surfaces. The calcaneum was then rotated, so as to bring its upper surface outward, till the internal made its appearance, when the soft structures were carefully separated, principally by the handle of the scalpel, thus completing the operation.

"After this mode of proceeding, we had the satisfaction of seeing the posterior tibial and plantar arteries pulsating in perfect integrity, while the tendons of the peroneal muscles, as well as those of the flexor longus pollicis, flexor communis digitorum, and tibialis posticus, were uninjured. No ligatures were required, the flap was approximated. A piece of flat sponge applied over the wound, and a gutta-percha splint previously moulded over the instep, so as to keep the foot extended. The operation was followed by a total cessation of pain and febrile action.

"The boy improved rapidly in health. The wound healed mostly by adhesion, the rest by granulation. The cavity partly filled up by what I suppose to be fibro-cellular tissue, and in two months after the operation the boy could run swiftly.

"At the present time he wears a small pad in the shoe under his heel, and does not experience the least inconvenience from the deficiency of the bone."

*A Case of Excision of the Hip-Joint for Morbus Coxarius, with remarks upon the propriety of such an operation, and a summary account of the recorded cases up to the present time.* R. A. KINLOCH, M.D., Surgeon to the Roper Hospital, and Lecturer on Surgery in the Charleston Summer Medical Institute. (Charleston Med. Journ., May, 1857, p. 307.)

"John McAllen, a native of Ireland, aged twenty, a laborer by occupation, was admitted into the Roper Hospital on the 2d of April, 1856. Two years previous he began to suffer from vague undefined pain about the lumbar region and the hip-joint of the right side. From time to time he had been compelled to lie up for a short period, but never, until nine months ago, had he been sufficiently unwell to be obliged to give up his usual avocations. More lately his attention had been attracted by a decided swelling beginning to manifest itself upon the front of his thigh. This had gone on increasing for several weeks, until it had attained its present volume. There was also a disposition, lately, for the thigh to become flexed upon the pelvis, and now the limb could not be entirely straightened. Upon examination of the patient, who was emaciated and presented a marked scrofulous appearance, I found a large fluctuating swelling, evidently sub-fascial, occupying nearly the whole anterior part of the middle third of the right thigh. The thigh was slightly flexed upon the pelvis, and the knee and foot turned a little in; the limb, generally, was emaciated. There was likewise a small swelling over the nates, behind and lower than the trochanter; this, like the larger swelling, did not in any way disappear on pressure. The swelling upon the thigh was what had given the patient most anxiety, and induced him to come to the hospital. He only wanted something done for this, as he felt well enough, as yet, to go about, and for aught else would not have been disposed to lie up. The diagnosis was at once made out as morbus coxarius. The swellings were considered to be chronic abscesses, but at first I could not determine if they communicated with the joint. The disease of the joint did not seem to have progressed very far, as the patient walked readily, and the joint functioned very well: no grating could be discovered on manipulation, nor did extensive movement give much pain. I delayed opening the abscesses for several days, in order to watch a little the constitutional power of my patient, and further, too, to have my diagnosis more certain. I prescribed for him cod-liver oil ℥ss. with wine ℥ij., three times a day, and an anodyne at night. April 10, the abscess of the thigh having materially increased in size, and the patient complaining of the feeling of tension, I made a kind of valvular opening at the dependent part, on the outer side of the sartorious muscle, and evacuated a large quantity of thin pus. April 22, I punctured the abscess over the nates, and, subsequently to this, both abscesses having refilled, were again opened on several occasions. May 4, distinct fluctuation was discoverable higher up, behind the trochanter. A puncture was made here, and considerable pus flowed away, mixed, it appeared, with synovial fluid. Consequent upon this there supervened considerable irritative fever, with gastric disturbance and great restlessness. The oil was now discontinued, and p. gum opii, gr. j., with a glass of wine, given every three hours. The distressing symptoms were modified after a couple of days, and the opium was then only given at bedtime. The discharge from the last abscess was now very copious and offensive. I had expressed a gloomy prognosis to some friends of the patient, and on this account I was applied to, on May 10, to give him a discharge from the hospital, his friends preferring that he should die at home. At their earnest solicitation, a few days after, I consented to visit him privately. At the expiration of a few days, I was much pleased to notice that a decided improvement had taken place under a free allowance of good wine, with nourishing diet, and opiates given when required. The discharge, though, continued profuse, and there were many evidences of extensive disease of the joint. Once or twice, when at the hospital, he heard me say something about dead bone and the persistence of the discharge. He now wanted to know if I could not in any way take out the dead bone, saying that he would submit to any operation that promised success. Eight or ten days elapsed, and finding that the discharge was as profuse as ever, moreover that ugly bed-sores were about appearing, I began to think that excision of the joint might offer a little chance. He seemed strong enough to stand an operation quickly performed, and this appeared the only alternative left. I then fairly stated to him my opinion, and told him how slight a chance the operation afforded; that it might hasten his end. He decided that the attempt should be made. I assumed the responsibility; but I determined that first I would explore the condition of the joint by making a free opening upon it through the cavity of a large abscess, just above the trochanter, and over the head of the femur. To open this abscess freely, I conceived to be proper practice, even though I did not intend to resect the joint. It would be but carrying out the improved plan of Mr. Gay for treating suppurating joints. If the capsule of the joint was found open, and the head of the bone caried, then I would proceed to resect. Previous to commencing my incisions, I had decided, by the test of Nélâton, that the head of the femur *was still in the acetabulum.* On the 31st of May, 1856, my patient having been brought under the influence of ether, (I used ether then instead of chloroform for the first and last time, at the suggestion of a friend; the patient took fully a pound before he became affected,) was turned slightly upon the left side, and held in that position by assistants. I then thrust the point of a small amputating knife a little in front of the base of the trochanter, and carrying it upward and outward, and then downward and backward, formed a semilunar incision encircling the trochanter, and consequently had a sort of semilunar flap with the convexity upward. The knife went easily through the walls of the abscess alluded to above, and exposed its entire cavity. The finger passed in, and to the bottom of the wound, discovered plainly considerable destruction of the capsule of the joint, and also the head of the bone, still in the acetabulum, but quite rough, and partially destroyed by caries; the brim of the acetabulum, too, could be felt considerably diseased. Under these circumstances, I conceived it best to proceed and resect the head of the femur. The point of the knife was accordingly passed across the portions of the capsule yet entire. My assistant then flexed and adducted the thigh, and, the round ligament not holding, the head was thrown out of the cavity, and then forced as much as possible through the external wound. I next passed the chain-saw behind the head and neck, and quickly divided the bone above the trochanters. Proceeding to examine the acetabulum, I was shocked at the extent of the disease. The brim was rough and crumbling, and there was an extensive perforation of the floor. With the gouge forceps I took away some portion of the brim, but soon desisted, as I felt it impossible to take away anything like the whole of the diseased structures. The wound was brought together by a few sutures, the lower end only kept open by a little lint to facilitate the exit of the discharge, the patient removed to bed, and the limb extended and kept steady by pillows. Not reacting as well as I desired, I ordered brandy freely until my afternoon visit, also a full dose of opium. In the afternoon his condition seemed better; he had warmth of skin, and a fair pulse. The brandy was continued, according to the indication, during the night, but upon my morning visit I was sorry to discover a greater disposition to collapse. From this time he refused to respond to the most active internal and external stimulation, but sank and died, not quite thirty hours after the operation. I was not able to examine the body after death."

In addition to the case in full, we quote the author's tables of forty cases, including three American, and conclude with a single paragraph in which Dr. Kinloch gives his own practical conclusions:—

TABLE OF CASES IN WHICH THE HIP JOINT HAS BEEN RESECTED FOR DISEASE.

| No. | Operator. | Sex and Age of Patient. | Duration of Disease. | Condition of Patient, and State of Parts at time of Operation. | Date of Operation, and Extent of Parts removed. | Progress and Result of Case. |
|---|---|---|---|---|---|---|
| 1 | Schmalz. | ......... | ................. | Caries; head disconnected from shaft. | 1816................................ | Recovered. |
| 2 | White. | M. 14. | three years. | Great exhaustion; head on dorsum of ilium; fistulæ. | 1818. Four inches of bone removed. | Fever slight; discharge little; health rapidly improved; well in a year; good use of limb. |
| 3 | Hewson. | ......... | ................. | Caries.......................... | 1823. Resected just above small trochanter. | Perforation of acetabulum. Died in three months. |
| 4 | Schlitching. | ......... | ................. | Caries, with abscess......... | 1829................................ | Well in six weeks; patient able to walk. |
| 5 | Kluge. | ......... | ................. | Caries head separated from shaft. | ..................................... | Died two months after operation. |
| 6 | Vogel. | Child. | ................. | Caries.......................... | ..................................... | Recovered. |
| 7 | Textor. | ......... | ................. | Disease of neck of bone and trochanter. | ..................................... | Sloughs formed on sacrum; death on fifty-third day. |
| 8 | Textor. | ......... | ................. | Caries; head out of acetabulum. | ..................................... | Gangrene of wound, and death on fourth day. |
| 9 | Heim. | ......... | ................. | ................................. | 1829................................ | Recovered. |
| 10 | Brodie. | ......... | ................. | Caries; head in acetabulum. | 1836................................ | Died in a few days. |
| 11 | Textor. | ......... | ................. | ................................. | 1845. Removed all above lesser trochanter. | Complete recovery. |
| 12 | Fergusson. | M. 14. | some months. | Head on dorsum; large sinus. | 1845. Four inches taken away. | Shock slight; wound healed well; health rapidly improved; limb quite useful. |
| 13 | Fergusson. | M. 8. | some time. | Emaciation and hectic; head dislocated; abscess over ilium; sinuses. | 1847. Head and neck with edges of acetabulum removed. | Health improved; wound never entirely healed; died, eight years after, of liver disease. |
| 14 | Roux. | M. 15. | longstanding. | Emaciation and hectic; dislocation, no abscess or fistulæ. | 1847. Head and part of neck removed. | Secondary hemorrhage; abscess between the glutei; death on seventh day; disease of acetabulum. |
| 15 | Simon. | Child. | two years. | Head dislocated upon acetabulum; abscesses and sinuses. | 1848. Head and portions of acetabulum removed. | Died four days after operation. |
| 16 | French. | F. 10. | some time. | Head carious, and upon dorsum of ilium. | 1848. Head and trochanter taken away; acetabulum healthy. | Recovered. |
| 17 | Fergusson. | ......... | ................. | ................................. | ..................................... | Recovered perfectly. |

| | | | | | | |
|---|---|---|---|---|---|---|
| 18 | Walton. | M. 16. | two years. | Caries; severe pain; sinuses with discharge; some disease of acetabulum. | 1848. Removed four inches of femur, and portions of acetabulum. | Pain ceased; health rapidly improved; wound healed well. |
| 19 | Walton. | ......... | .................. | .................................. | 1848.................................. | Unsuccessful. |
| 20 | Smith. | M. 33. | year and half. | Head dislocated; caries; sinuses. | 1848. Removed three inches and rim of acetabulum. | Symptoms improved for four months, but in two weeks more he died; Bright's disease of kidney, and caried vertebræ found. |
| 21 | Fergusson. | F. 13. | three years. | Caries; head dislocated. | 1849. Removed head and trochanter major. | Recovered perfectly. |
| 22 | Morris. | M. 18. | six years. | Neck nearly destroyed; head not dislocated. | 1849. Head and neck removed. | Recovered, with motion of thigh, and could walk a short distance. |
| 23 | Cotton. | F. 12. | .................. | Ulcer upon hip; caries; sinuses; emaciation. | 1849. Four and a half inches removed. | Slight fever; symptoms soon improved; ulcers healed; health gaining. |
| 24 | Buchanan. | M. 41. | two years. | Great suffering; grating in joint; profuse discharge. | 1850. Resected below trochanter; head and edge of cavity carious. | Recovering rapidly, but died of dysentery three months after; parts found healthy and in advanced stage of repair. |
| 25 | Skey. | F. 13. | three years. | Emaciated and nearly worn out; femur dislocated on dorsum; large ulceration on trochanter; sinuses discharging freely. | 1850. Head found absorbed; portion of neck and the trochanter removed. | Result not mentioned. |
| 26 | Sayre. | ......... | .................. | Caries of head and neck. | 1851. Head removed. | Wound healed, but abscesses formed; did not progress favorably. |
| 27 | Jones. | F. 32. | 18 years. | Powers of life feeble; head carious, and on dorsum; sinuses. | 1851. Head removed. | Recovered; walks some, with a high heel and a stick. |
| 28 | Stanley. | M. 18. | several years. | Feeble and emaciated; head dislocated; fistulæ discharging freely. | 1852. Large abscess found under glutei. | Symptoms improved rapidly; wound not healed entirely, but health robust eighteen months after operation. |
| 29 | Hawkins. | F. 10. | four years. | Head carious; emaciation and hectic. | 1852. Removed one inch below the trochanter, also edges of acetabulum. | Died on third day; found large perforation of acetabulum. |
| 30 | Bigelow. | M. 10. | .................. | Caries and dislocation. | 1852. Bone separated while sawing. | Death on the twelfth day. |
| 31 | Erichsen. | M. 14. | several years. | Greatly reduced; bone dislocated. | 1853. Neck and great trochanter removed. | Rapid improvement; suppuration profuse; died some months after. |

| No. | Operator. | Sex and Age of Patient. | Duration of Disease. | Condition of Patient, and State of Parts at time of Operation. | Date of Operation, and Extent of Parts removed. | Progress and Result of Case. |
|---|---|---|---|---|---|---|
| 32 | Parkman. | M. 12. | six months. | Hectic; abscess; caries. | 1853 Head and part of neck removed. | Three months after, progress very satisfactory. |
| 33 | Fergusson. | F. 13. | one year. | Head dislocated; abscess. | 1854. Removed head and large trochanter. | Improvement marked. |
| 34 | Erichsen. | M. 8. | long standing. | Emaciation; hectic; caries. | 1854. Removed head and edges of acetabulum. | Recovered. |
| 35 | Sayre. | F. 9. | 18 months. | Caries; dislocation on dorsum; abscesses; great prostration; sweats, &c. | 1854. Removed head and neck; gouged away portion of brim of acetabulum. | Recovered, with limb only quarter of an inch short; flexion, extension, and rotation preserved; general health perfect. |
| 36 | Shaw. | M. 17. | 7 or 8 months. | Dislocation and protrusion of head through fistulous opening. | 1856. Head removed. | Recovery, with a very useful limb; can walk three miles at a time. |
| 37 | Shaw. | F. 14. | two years. | Dislocation; fistulæ; sinuses. | 1856. Head and neck removed. | Excellent condition thirteen weeks after the operation. |
| 38 | Hancock. | M. 14. | five yeass | Caries, with abscess discharging and connecting with interior of the pelvis. Probe passes from a fistulous opening in the groin into the pelvis, and then out through the acetabulum; great emaciation; night sweats and cough, with expectoration streaked with blood. | 1856. Resected below great trochanter; also removed the whole floor of the acetabulum. | Patient hopping about on crutches sixteen weeks after the operation, with robust health. |
| 39 | Erichsen. | M. | ................ | ........................... ...... | 1857. Removed head. | One month after the operation, the patient is doing well. |
| 40 | DeMorgan. | M. 17. | ................ | Caries of head; dislocation. | 1857. Head and neck removed. | Little constitutional disturbance; patient doing well. |

NOTE.—After the materials for the above table had been gathered, I for the first time saw the article of Dr. Sayre, of New York, in the 14th vol. of the New York Journal of Medicine. I was pleased with his table, and so made my own conform to his arrangement in part. One or two cases recorded by him I have not been able to find reports of anywhere else, so these must rest upon his authority. I have no doubt of their correctness, and it is likely that I have overlooked them in my examination of the various periodicals in my possession. I present in my list several cases omitted by Dr. Sayre; and writing two years after him, have of course recorded many which have recently occurred.—R. A. K.

"The above record proves the mortality to be much less than can be shown for amputation at the joint; and so far as it pronounces upon the utility of the limb after excision, the verdict is satisfactory."

Dr. Kinloch's creed upon the question of operating in hip-joint disease may be learned from its expression in the following words:—

"To explain, then, more accurately my views as to the propriety of excision of the hip-joint for disease, I would say that so soon as I could discover the existence of abscess of the joint, I should *entertain the prospect of the operation.* The late researches of Mr. Gay and others have fully established the advantages of making free openings into suppurating joints. This should be the practice, then, thus early in the disease. Afterward, the usual treatment for improving the general health, and for securing absolute rest to the joint, should be persevered in. If no improvement of health, and no diminution of the discharge took place within a reasonable time, I should consider that the greatest amount of security was offered by excision. I do not mean to say that recovery would not, under these circumstances, take place in many instances without operation; but I think that it would, upon the whole, be more doubtful,—I should have more faith in surgery than in the *vis medicatrix naturæ.* I advocate an early operation, because delay only lessens the chances of success; but I likewise am in favor of action in very late stages of the disease, as must be seen from my course in the case which has led to these remarks. But here *the operation is, properly speaking, an experimental one.* To my mind, however, it is justifiable, because I must believe that it will seldom be instrumental in shortening life. If the patient is not too reduced to stand the shock of an operation, I think it affords him a little chance, even though disease may be so extensive as not to permit of its entire removal. If the pelvic portion of the joint has suffered extensively, and cannot be entirely removed, its abnormal action is more likely, as already intimated, to be arrested than increased by the operation."

*Compound Dislocation of the Long Bones, considered with especial reference to the value of Resection.* FRANK HASTINGS HAMILTON, M.D., Professor of Surg. Med. Dep. University of Buffalo. (American Journal of the Medical Sciences, Oct. 1857, p. 324.)—This subject is discussed with characteristic clearness and thoroughness in an elaborate paper, in which two cases are given in illustration. We have room only for the concluding remarks.

"Thus, if we consider the question of the life of the patient only, the argument and the testimony seem to favor resection in a great majority of cases of compound dislocations occurring in large joints, and in a considerable number of cases of similar accidents in the smaller joints. It is certainly more safe than non-reduction or reduction without resection, and it is probably quite as safe as amputation or tenotomy.

"But there is another question, which is, in our estimation, secondary to the one now considered, but which is often, in the estimation of the patient himself, of the first importance—namely, by which method will he suffer the least maiming or mutilation?

"This question I do not find it difficult to answer. Certainly it is not by non-reduction or by amputation; and, putting tenotomy aside, it is now a question only between reduction without resection, and reduction with resection. These two methods, one of which experience has shown to be fraught with danger, and the other of which experience has shown to be relatively safe, are now to be compared in a point of view in which their antagonisms are perhaps less conspicuous, yet sufficiently marked.

"*First.* In either case the inflammation consequent upon the injury may be violent, and the recovery slow and tedious. The same arguments, however, which we have applied to the question of the comparative danger of the two modes, must apply with nearly equal force to this question of maiming; since the amount of maiming must often be governed by the intensity and duration of the inflammation, and upon this point the testimony has been shown to be in favor of resection.

"It will be observed that not only is the danger of maiming rendered more considerable by reduction without resection, because the inflammation is so much more likely to extend to the tendons and muscles, causing them to adhere to each other, and to become subsequently atrophied, a condition from which they often never completely recover, but also because the ligaments and capsules of the joints, with the synovial surfaces, are in consequence encroached upon, and the freedom of motion is ever afterwards greatly restricted, if not completely lost. This marked impairment of the functions of the joint does not always happen, but it cannot be denied that it does generally. Indeed it is by no means uncommon for these accidents to be followed, after ulcerations of the cartilage, by copious bony deposits in and around the joints.

"How is it, on the other hand, with these joints after resection? I have thus far heard of no cases in which complete anchylosis resulted; but in all considerable freedom of motion has returned, and in some the restoration in this respect has been nearly or quite as complete as before the accident.

"Says Dr. Kerr, of Northampton:—

"'Several cases of compound dislocation of the ankle have fallen under my care, and it has been uniformly my practice to take off the lower extremity of the tibia, and to lay the limb in a state of semiflexion upon splints; by this means a great deal of painful extension, and the consequent high degree of inflammation, are avoided. The splints I used are excavated wood, and much wider than those in common use, with thick movable pads stuffed with wool. I keep the parts constantly wetted with a solution of liquor ammoniæ acetatis, without removing the bandage. In my very early life, upward of sixty years ago, I saw many attempts to reduce compound dislocations without removing any part of the tibia; but, to the best of my recollection, they all ended unfavorably, or, at least, in amputation. By the method which I have pursued, as above mentioned, I have generally succeeded in saving the foot, and in preserving a tolerable articulation.'*

"Sir Astley Cooper has made a valuable experiment to determine the condition of the new joint under these circumstances; and the vast number of cases in which resection has now been practiced in cases of caries of the articulating surfaces, and their results, add still more substantial proofs as to the usefulness of the joints after such operations.

"'I made an incision upon the lower extremity of the tibia, at the inner ankle of a dog, and cutting the inner portion of the ligament of the ankle-joint, I produced a compound dislocation of the bone inward. I then sawed off the whole cartilaginous extremity of the tibia, returned the bone upon the astragalus, closed the integuments by suture, and bandaged the limb to preserve the bone in this situation. Considerable inflammation and suppuration followed; and in a week the bandage was removed. When the wound had been for several weeks perfectly healed, I dissected the limb. The ligament of the joint was still defective at the part at which it had been cut. From the sawn surface of the tibia there grew a ligamento-cartilaginous substance, which proceeded to the surface of the cartilage of the astragalus, to which it adhered. The cartilage of the astragalus appeared to be absorbed only in one small part; there was no cavity between the end of the tibia and the cartilaginous surface of the astragalus. A free motion existed between the tibia and astragalus which was permitted by the length and flexibility of the ligamentous substance above described, so as to give the advantage of a joint where no synovial articulation or cavity was to be found. This experiment not only shows the manner in which the parts

* Cooper on Dis. and Fract., p. 275.

are restored, but also the advantage of passive motion; for if the part be frequently moved, the intervening substance becomes entirely ligamentous; but if it be left perfectly at rest for a length of time, ossific action proceeds from the extremity of the tibia into the ligamentous substance, and thus produces an ossific anchylosis.'*

"*Second.* Is it not probable, moreover, since the limb can be retained in place so much more easily after resection, that it will actually, in a majority of cases, be found to have been retained in place more perfectly? Even after simple dislocations, especially in those occurring at the ankle-joint, great deformity and much maiming are the not unfrequent results, and that too when all diligence and care have been employed. It has been impossible always to maintain a perfect apposition in the articulating surfaces. How much greater must be this difficulty in cases of compound dislocations!

"*Third.* The only argument which remains in favor of reduction without resection is the necessary shortening of the limb after resection. But this need seldom perhaps to exceed three-quarters of an inch, and often not more than half an inch; an amount of shortening which, as I have had occasion to prove when treating of fractures, does not necessarily produce a halt, and which indeed is often not known to exist by the patient himself.

"*Finally,* it must not be inferred that the writer intends to recommend resection as a universal practice in cases of compound dislocation of the long bones. He has only sought to determine in a general manner its relative value as compared with other modes of procedure; and especially has it been his intention to bring more prominently into view the importance of rest and relaxation to the muscles, as an element in the treatment most essential to success. To declare its special application to cases, would demand a treatise more elaborate than it was proposed to write. If, however, one were to speak of the individual bones only, there seems sufficient authority in the facts and arguments already presented to conclude that resection is applicable to certain compound dislocations of the clavicle, humerus, radius and ulna, fingers, femur, tibia and fibula, and toes; in short, to all of these accidents occurring in the long bones of the extremities."

*Successful Amputation at the Hip-Joint.* GEO. C. BLACKMAN, M.D., Professor of Surgery in the Medical College of Ohio; Surgeon to the Commercial Hospital, Cincinnati. (Western Lancet, Jan. 1857, p. 7.)

"John Pfiel, a German, twenty years of age, was admitted into the Commercial Hospital, November, 1855. He complained of an enlargement of the lower part of the femur, which somewhat interfered with locomotion, but caused him but little pain. The tumor, evidently of an osseous character, was oblong in shape, and extended from just above the condyles to about the middle of the thigh. The integuments were of natural appearance. In the course of from four to five weeks, the tumor was found to have rapidly increased in size, and bid fair soon to involve the whole of the femur. In a clinical lecture upon the case, we expressed our fears that the disease was of a malignant character, and gave an unfavorable prognosis. At the same time, the patient's health being tolerably good, we proposed amputation at the hip-joint as the only alternative. To this he assented, and on the 8th of December, 1855, in the presence of the class, we disarticulated the thigh, assisted by Prof. T. Wood, Dr. Thompson, of Columbus, Dr. Fries, of Cincinnati, Dr. Evans, of Covington, and Dr. Rae, the House surgeon. The operation adopted was that by the anterior and posterior flaps, and was completed within thirty seconds. On examination, the femur was found to be affected with osteo-cephaloma, the disease involving two-thirds of the shaft of the bone. As the patient was under the influence of a mixture of chloroform and sulphuric ether, he suffered no pain, but was greatly pros-

* Cooper on Dis. and Fract., p. 281.

trated—although the hemorrhage, owing to the skilful compression by Prof. Wood, was slight. For several days the pulse ranged from 130 to 160 per minute; and it was not until after the fourth day that reaction was satisfactorily established. The greater portion of the surfaces of the flaps united by the first intention; but some sinuses continued for several months, and even to this day (Dec. 22, 1856,) there are two or three fistulous openings, which probably communicate with the cotyloid cavity. During the month of September last, the patient suffered severely from a dysenteric affection, and for weeks was in a very feeble state. For some time past he has been in the City Infirmary, near Carthage, and we have been informed within the past ten days by the physician of the Institution, Dr. Lassing, that although not yet fully recovered from the attack to which we have alluded, there are no appearances of a return of the disease for which he submitted to amputation at the hip-joint. It will be remembered that this report is written 54 weeks after the operation. The treatment of the case, for weeks after the operation, was under the skilful superintendence of Dr. Rae, and to his unremitting care the patient is undoubtedly indebted for his recovery."

Dr. Blackman's paper furnishes, in conclusion, a valuable summary sketch of the statistics of the operation.

For want of space we are compelled to postpone the remainder of this Report to the July Number. It will appear in the form of a Second Part.

